# Psychological Distress and Biological Stress Markers After Spinal Cord Injury: A Longitudinal Multi-Omics Pilot Study

**DOI:** 10.3390/ijms27156574

**Published:** 2026-07-23

**Authors:** Simona Capossela, Alessandro Bertolo, Alexander Stacul, Franziska Singer, Claudio Peter, Xavier Jordan, Margret Hund-Georgiadis, Björn Zörner, Jivko Stoyanov

**Affiliations:** 1 SCI Population Biobanking and Translational Research, Swiss Paraplegic Research, 6207 Nottwil, Switzerland; simona.capossela@paraplegie.ch (S.C.);; 2 Institute of Social and Preventive Medicine, University of Bern, 3012 Bern, Switzerland; 3 NEXUS Personalized Health, ETH Zurich, 8092 Zurich, Switzerland; 4 SIB Swiss Institute of Bioinformatics, 1015 Lausanne, Switzerland; 5 Department of Health, ZHAW Zurich University of Applied Sciences, 8400 Winterthur, Switzerland; 6 Clinique Romande de Réadaptation, 1951 Sion, Switzerland; 7 REHAB Basel, 4055 Basel, Switzerland; 8 Swiss Paraplegic Center, 6207 Nottwil, Switzerland

**Keywords:** spinal cord injury, psychological distress, inflammation, aging biomarkers, transcriptomics, proteomics

## Abstract

This pilot study examined longitudinal changes in psychological distress and biomarkers related to stress, inflammation, and biological aging during the first inpatient rehabilitation after Spinal Cord Injury (SCI). We analyzed data from 120 participants in the SwiSCI inception cohort at two timepoints: the beginning of the first post-injury rehabilitation and discharge. A subset of 50 participants representing four distress patterns (high chronic, low, transient, increasing distress) underwent biomarker analyses, including measurements of immune and inflammatory proteins and whole-blood transcriptomic profiling. The results showed that psychological distress, depression, and protein carbonyl content decreased significantly by discharge. In contrast, cortisol levels and telomere length showed no significant changes. Proteomic analyses revealed a broad downregulation of inflammatory proteins (e.g., IFN-γ, IL-10, LIF) at discharge, whereas transcriptomic profiles did not differ between timepoints. The high chronic distress group showed lower expression of six genes involved in adaptive immunity, neural development and apoptosis. Psychological distress was not linearly associated with global biomarker profiles. However, CXCL1 and IL-7 showed low-to-moderate correlations with depression and anxiety, respectively. This pilot study demonstrates the feasibility of integrating psychological and molecular data to investigate biological response following SCI. The findings are exploratory and need validation in larger studies with longer follow-up periods.

## 1. Introduction

Spinal cord injury (SCI) is a chronic, life-altering condition that often leads to marked physical disability, long-term health complications, and a considerable psychosocial burden, resulting in significant impairments in mobility, autonomy, and quality of life [1,2]. Beyond the primary neurological lesion, SCI is associated with a wide spectrum of chronic secondary complications, including cardiovascular dysfunction, musculoskeletal deterioration, metabolic syndrome, immune dysregulation, recurrent infections, and respiratory impairment [3]. Alongside these physical challenges, individuals living with SCI frequently experience psychological distress, characterized by elevated levels of stress, depression, and anxiety [4,5].

Individuals with SCI often experience accelerated or premature aging, whereby age-related physical and physiological changes occur earlier and progress more rapidly than in the general population [6,7]. Aging in individuals with SCI is accompanied by symptoms such as fatigue, chronic pain, weakness, and spasticity, as well as by a cluster of pathological conditions, including cardiovascular disease, metabolic disorders, osteoporosis, respiratory conditions, and cancer [8]. These complications contribute to reduced activity, loss of independence, and social isolation, all of which substantially increase psychological distress and vulnerability to additional comorbidities. Recent studies have reported that 30–33% of individuals with SCI experience depression and/or anxiety at the time of rehabilitation discharge [9,10]. The development of mood disorders in this context is influenced by multiple factors, including the initial trauma, concerns about secondary health conditions, individual predisposition, and uncertainty regarding the future [8,11]. Interestingly, injury level and the severity of functional impairments are not consistently associated with the degree of psychological distress, suggesting that other factors, including physiological changes induced by SCI, may play an important role [12]. Psychological distress has been associated with accelerated biological aging through hormonal dysregulation, inflammation, oxidative stress, and cellular damage, thereby increasing the risk of age-related diseases and accelerating biological decline [13,14,15,16].

An important physiological mediator of stress-related outcomes is the neuroendocrine response. During periods of persistent distress, the hypothalamic–pituitary–adrenal (HPA) axis is repeatedly activated, leading to the release of stress hormones such as cortisol. Cortisol plays a central role in coordinating the body’s response to psychological and physiological challenges by mobilizing glucose to meet energy demands and supporting tissue repair processes [17]. In parallel, biochemical indicators such as protein carbonyl content (PCC) provide insight into stress responses at the cellular level. PCC reflects the irreversible oxidation of amino acid residues in proteins and is widely used as a marker of oxidative stress [18]. The accumulation of oxidative damage is closely linked to aging [15] and the development of age-related diseases such as diabetes [19]. Together, cortisol and PCC provide complementary perspectives on stress: cortisol reflects systemic hormonal responses, whereas PCC captures molecular and cellular consequences, showing how prolonged psychological distress may translate into measurable biological changes.

Prolonged stress responses can result in sustained elevations of glucocorticoids and pro-inflammatory cytokines, promoting a state of chronic systemic inflammation [20]. This persistent low-grade inflammation is increasingly recognized as a key driver of age-related diseases, including cardiovascular, musculoskeletal, respiratory, and neurodegenerative diseases [8,16,21,22]. Emotional distress has also been associated with reduced telomere length, a widely accepted biomarker of cellular aging. Shortened telomeres are linked to increased susceptibility to degenerative diseases, higher mortality, and reduced tissue regenerative capacity [23]. Clinical characteristics such as reduced mobility and bladder dysfunction, which are common among individuals with more severe SCI, have likewise been associated with shorter telomere length [24].

This pilot feasibility study aimed to explore the relationship between psychological distress and changes in stress- and aging-related biomarkers among individuals with SCI during their first inpatient rehabilitation after injury. Using a longitudinal cohort design, we examined changes in cortisol, PCC, telomere length, and inflammatory biomarkers during rehabilitation and investigated their association with self-reported psychological distress.

## 2. Results

### 2.1. Study Population

Within the Swiss Spinal Cord Injury (SwiSCI) Cohort Study, data were collected from 120 patients with traumatic and non-traumatic SCI, including etiologies such as multiple sclerosis, amyotrophic lateral sclerosis and polyneuropathy. Participants completed the SwiSCI questionnaire at four timepoints and donated blood samples to the SwiSCI Biobank at the beginning (T1) and end (T4, discharge) of their first inpatient rehabilitation period. Patients received various medications during rehabilitation, including psychotropic drugs (antiepileptics, psycholeptics, and antidepressants), analgesics, and non-steroidal anti-inflammatory drugs (NSAIDs). By discharge (T4), the proportion of patients receiving psychotropic medications and analgesics decreased by 12% and 36%, respectively, whereas NSAIDs use remained stable at 15% across both timepoints.

For the biological analyses, a subset of 50 patients was selected to represent four distinct patterns of self-reported psychological distress during rehabilitation (high chronic, low, transient, and increasing distress). This subgroup did not differ significantly from the full cohort in terms of clinical characteristics. Clinical and demographic data, together with the statistical comparison between the two groups, are summarized in Table 1.

### 2.2. Distress Thermometer, Depression and Anxiety Scores

**Figure 1:**
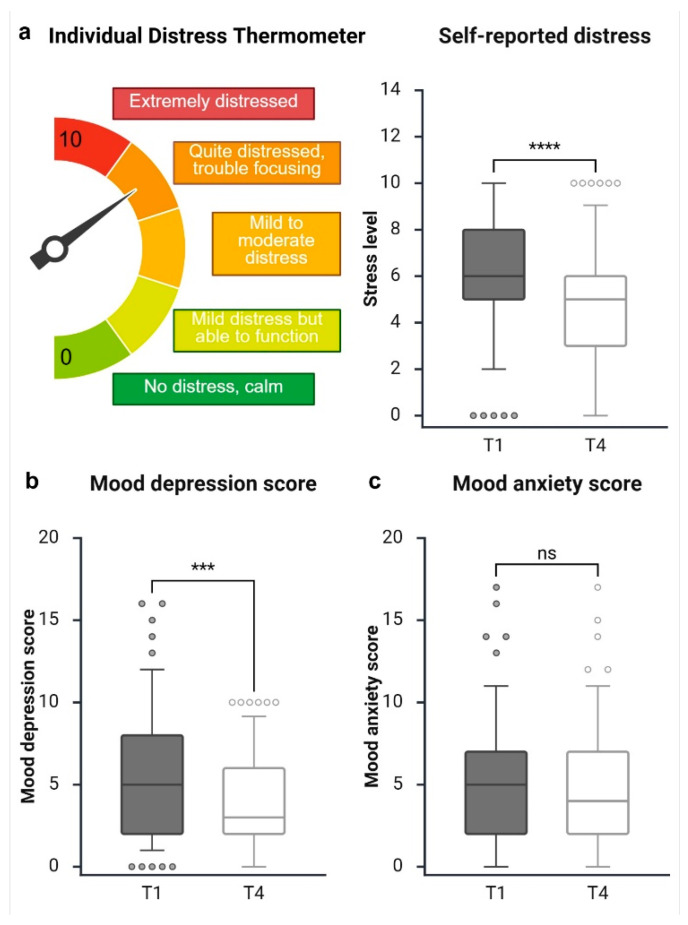
Psychological distress and mood outcomes in individuals with SCI at T1 and T4 (**a**) Self-reported distress levels, as measured by the Distress Thermometer, decreased significantly from T1 to T4. (**b**) Depression scores were significantly reduced at T4 compared with T1. (**c**) Anxiety scores showed no significant change between T1 and T4. *n* = 120; *** *p* < 0.001, **** *p* < 0.0001, ns = not significant. Created in BioRender. Bertolo, A. (2026). https://BioRender.com/8gkf8bv.

The Distress Thermometer showed that individuals with SCI (*n* = 120) remained generally distressed at the end of the first rehabilitation period. However, psychological self-reported distress decreased significantly following rehabilitation (*p* < 0.001), with median scores decreasing from 6 (IQR 3) at T1 to 5 (IQR 3) at T4 (Figure 1a).

Depression scores significantly decreased at T4 (median 3; IQR 4) compared with T1 (median 5; IQR 6) (*n* = 118; *p* < 0.001; Figure 1b), whereas anxiety scores did not differ significantly between timepoints (T1: median 5, IQR 5; T4: median 4, IQR 5; *n* = 118; Figure 1c).

Overall, no linear correlation was found between self-reported distress and age, gender, type of paralysis (paraplegia or tetraplegia) or type of injury (traumatic or non-traumatic). In contrast, moderate positive linear correlations were observed between self-reported psychological distress and both depression (Pearson’s r = 0.58) and anxiety scores (Pearson’s r = 0.45).

### 2.3. Distress Directional Change Patterns (T1–T4)

**Figure 2:**
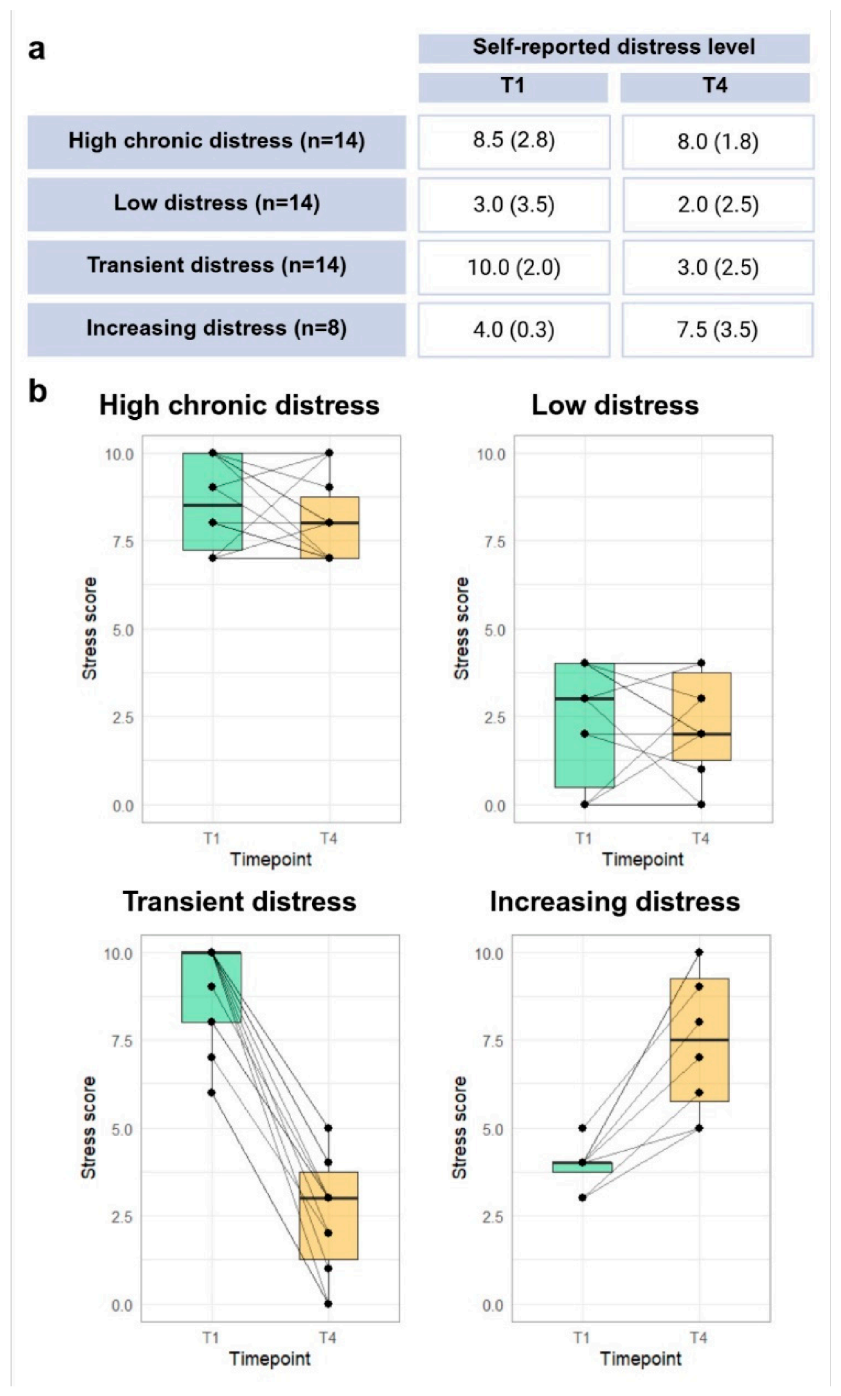
Distress groups of individuals with SCI included in the biological analyses during the first rehabilitation period (T1–T4). (**a**) Participants were classified into four distress groups based on the direction of change in self-reported Distress Thermometer scores between T1 and T4: high chronic, low, transient and increasing distress. Values are presented as median (IQR). (**b**) Individual changes in Distress Thermometer scores within each group. The high chronic group maintained elevated distress at both timepoints, whereas the low distress group consistently reported low distress. Participants with transient distress showed high distress at T1 followed by a marked reduction at T4, whereas those with increasing distress exhibited the opposite pattern. Green and yellow boxplots represent T1 and T4, respectively. Created in BioRender. Bertolo, A. (2026). BioRender.com/mhutto1.

For subsequent biological analyses, we selected a subset of 50 individuals with SCI representing four distinct patterns of self-reported psychological distress during the first rehabilitation period following injury (T1–T4) (Figure 2a,b): “high chronic distress”, defined as distress levels at and above 7 at both timepoints; “low distress”, defined as distress levels at and below 4 at both timepoints; “transient distress”, characterized by decreasing distress from T1 to T4; and “increasing distress”, characterized by increasing distress from T1 to T4.

In the SwiSCI cohort study, self-reported distress was also assessed at two intermediate timepoints during rehabilitation (T2 and T3). However, because of prolonged stays in intensive care after injury or early discharge, distress data were not consistently available across all timepoints. In the present study, self-reported distress data were available for all participants at T1 and T4 (*n* = 50), but only for 58% of participants at T2 (*n* = 29) and 44% at T3 (*n* = 22). Distress directional change patterns across all four timepoints are shown in Supplementary Figure S1.

### 2.4. Stress- and Aging-Related Biomarkers

Stress- and aging-related biomarkers, namely cortisol, protein carbonyl content (PCC), and telomere length, were measured at T1 and T4 in the subset of individuals with SCI included in the biological analyses (*n* = 50). Comparisons were performed between the two timepoints (T1 vs. T4) and across the four distress groups (high chronic, low, transient and increasing distress).

**Figure 3:**
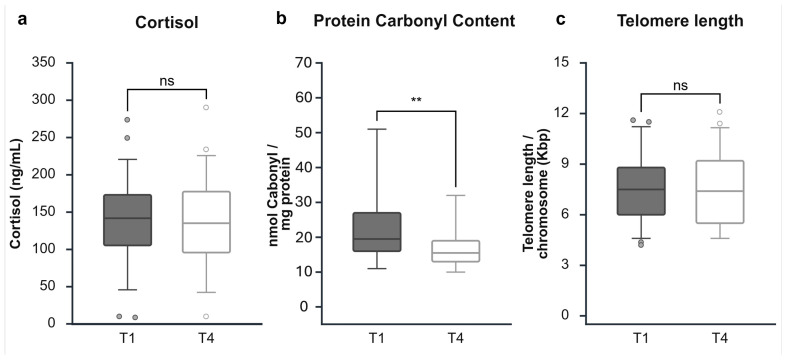
Stress- and aging-related biomarkers in individuals with SCI at T1 and T4. (**a**) Serum cortisol concentrations showed no significant difference between T1 and T4 (*n* = 48). (**b**) Oxidative stress, measured by protein carbonyl content, was significantly reduced at T4 compared with T1 (*n* = 48). (**c**) Telomere length per chromosome end did not differ significantly between timepoints (*n* = 49). Significance was determined by paired statistical tests: ** *p* < 0.01; ns, not significant. Created in BioRender. Bertolo, A. (2026). https://BioRender.com/rbbydys.

Serum cortisol levels ranged from 9 to 290 ng/mL (median 141 ng/mL, IQR 73; *n* = 48). No significant difference was observed between T1 (median 142 ng/mL, IQR 68) and T4 (median 135 ng/mL, IQR 82) (Figure 3a).

A significant decrease in PCC (*p* < 0.01; *n* = 48) was observed at T4 (median 15 nmol/mg protein, IQR 0.6) compared with T1 (median 20 nmol/mg protein, IQR 1.1) (Figure 3b).

Telomere length was measured in blood DNA (*n* = 49), and no significant difference was found between T1 (median 7.5 kbp, IQR 2.8) and T4 (median 7.4 kbp, IQR 3.7) (Figure 3c).

Although telomere length was negatively correlated with age (Pearson’s r = −0.7), none of the three biomarkers showed a linear correlation with self-reported psychological distress or mood outcomes.

### 2.5. Transcriptome Analyses

Transcriptomic analyses were performed on RNA extracted from blood collected from a subset of 50 individuals with SCI selected for biological analyses at both timepoints (T1 and T4). Quality control of FASTQ files revealed technical issues during sequencing preparation, namely incomplete hemoglobin depletion, resulting in a high proportion of hemoglobin-derived reads. Consequently, gene expression counts were dominated by hemoglobin-related genes. Residual hemoglobin transcript content varied considerably across samples, ranging from 2% to 80%, resulting in a batch effect. To mitigate this issue, samples were re-sequenced to yield more counts and hemoglobin-related gene counts were removed prior to downstream analyses.

After these correction steps, no significant linear correlation was observed between hemoglobin content and psychological distress levels at each time point (one-way ANOVA, *p*-value = 0.89).

**Figure 4:**
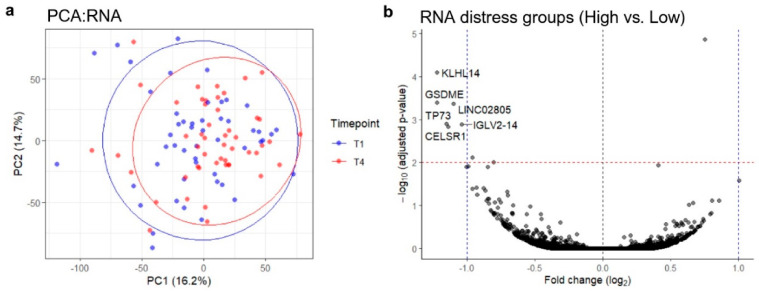
Transcriptomic analyses in individuals with SCI at T1 and T4 (**a**) Principal component analysis (PCA) of blood transcriptomes at T1 (blue) and T4 (red), illustrating the clustering of samples across the two timepoints. (**b**) Volcano plot displaying differentially expressed genes between the high chronic and low distress groups, highlighting IGLV2-14, CELSR1, GSDME, KLHL14, TP73, and LINC02805 as significantly downregulated genes. The blue dashed vertical lines indicate log_2_ fold change thresholds of −1 and +1, the gray dashed vertical line indicates no change in gene expression (log_2_ fold change = 0), and the red dashed horizontal line indicates the significance threshold corresponding to an adjusted *p* value of 0.01 (−log_10_ adjusted *p* value = 2). Created in BioRender. Bertolo, A. (2026). https://BioRender.com/sqmqnux.

Several comparisons were made, including analyses between the two timepoints (T1 vs. T4) and among the four distress groups (high chronic, low, transient and increasing distress). As shown in the principal component analysis (PCA) plot (Figure 4a), transcriptomic profiles did not show a clear separation between T1 and T4, suggesting that no significant changes in global gene expression occurred during rehabilitation.

In contrast, the comparison between the high chronic and low distress groups identified six significantly downregulated genes in the high chronic distress group (Figure 4b and Supplementary Table S1). These included genes associated with immune function, neural development, programmed cell death, tumor suppression, and gene regulation (IGLV2-14, CELSR1, GSDME, KLHL14, TP73, and the long intergenic non-coding RNA LINC02805). Consistent with the differential expression results, Gene Set Enrichment Analysis (GSEA) comparing the high chronic and low distress groups revealed reduced activity of pathways involved in immune responses in the high chronic distress group, including pathways related to adaptive immunity (B-cell signaling), innate immune effector responses (Fc receptor signaling and phagocytosis), and complement-mediated inflammation (Supplementary Table S2).

### 2.6. Inflammatory Protein Profile (Proteomic Analyses)

Proteomic analyses were performed on serum samples from individuals with SCI (*n* = 48) collected at both timepoints (T1 and T4) using Olink Targeted Proteomics. Quality control of proteomic data showed that all samples passed the internal Olink quality control criteria (Supplementary Figures S2 and S3) and all proteins except IFNA2 were successfully detected across samples (Supplementary Figures S4 and S5). Inter- and intra-plate coefficients of variation indicated high assay reproducibility (Supplementary Figure S6), while inter-run and intra-run coefficients of variation further confirmed the robustness of the measurements (Supplementary Table S3).

**Figure 5:**
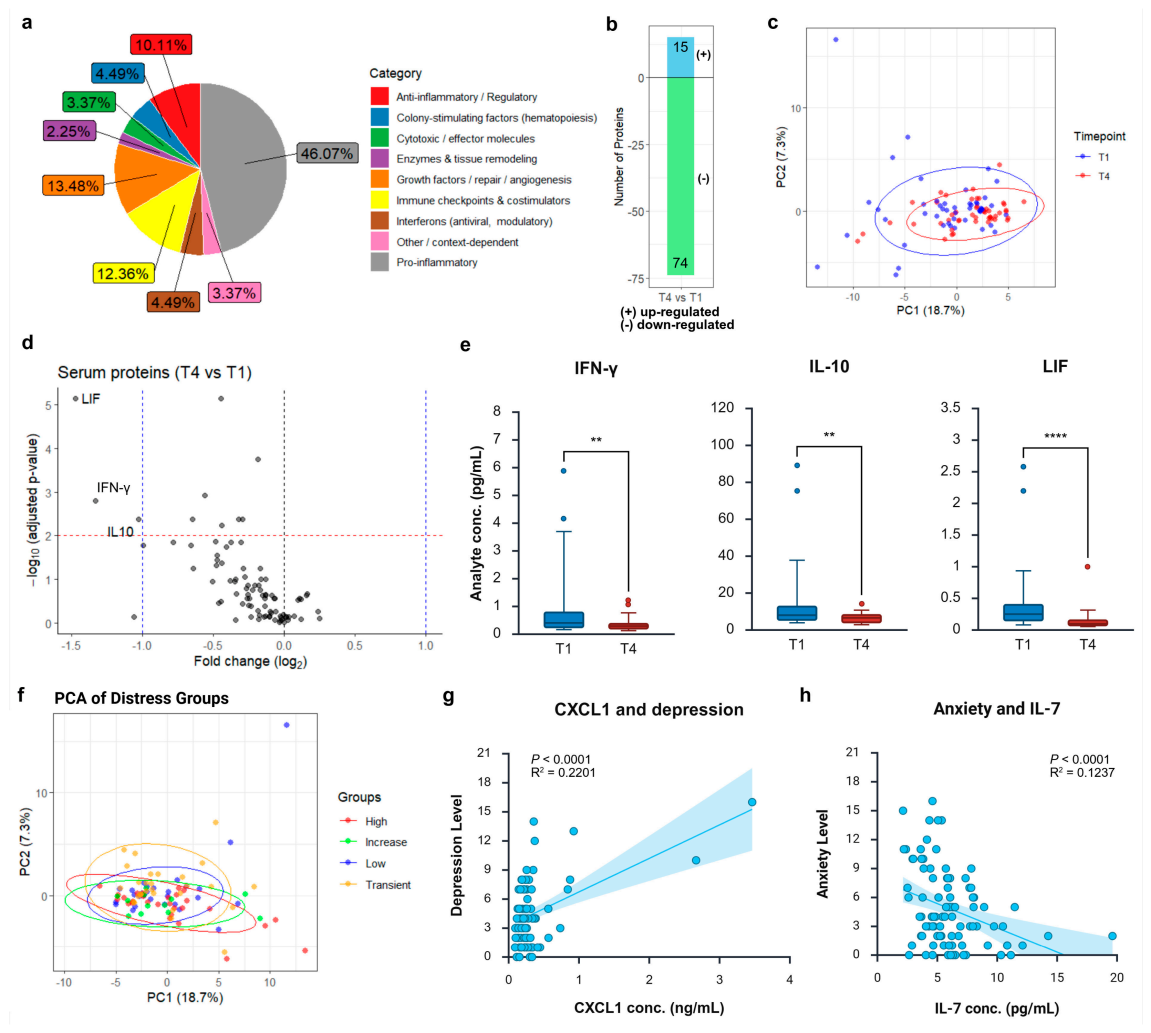
Proteomic profiling of serum samples across timepoints (T1 and T4), psychological distress, and mood outcomes in individuals with SCI. (**a**) Distribution of detected serum proteins according to functional categories, showing a predominance of anti-inflammatory and regulatory factors. (**b**) Summary of differential protein expression between T4 and T1, indicating 15 upregulated and 74 downregulated proteins. (**c**) Principal component analysis (PCA) of serum proteomes at T1 (blue) and T4 (red), illustrating the clustering of samples across the two timepoints. (**d**) Volcano plot displaying differentially expressed proteins between T4 and T1, highlighting LIF, IFN-γ, and IL-10 as significantly altered proteins. The blue dashed vertical lines indicate log_2_ fold change thresholds of −1 and +1, the gray dashed vertical line indicates no change in gene expression (log_2_ fold change = 0), and the red dashed horizontal line indicates the significance threshold corresponding to an adjusted *p* value of 0.01 (−log_10_ adjusted *p* value = 2). (**e**) Box plots showing serum concentrations of IFN-γ, IL-10, and LIF at T1 and T4 (**, *p* < 0.01; ****, *p* < 0.0001). (**f**) PCA of serum proteomes stratified by distress group (high chronic, increase, low, transient distress), showing similar proteomic profiles across groups. (**g**) Positive correlation between serum CXCL1 concentration and depression scores (*p* < 0.0001, R^2^ = 0.2201). (**h**) Negative correlation between serum IL-7 concentration and anxiety scores (*p* < 0.0001, R^2^ = 0.1237). Statistical analyses were performed using adjusted *p*-values for multiple comparisons (*n* = 48). Created in BioRender. Bertolo, A. (2026). https://BioRender.com/27bcmu3.

A total of 89 inflammatory and immune-related proteins (Figure 5a) were measured. Comparisons were performed between the two timepoints (T1 vs. T4) and across the four distress groups (high chronic, low, transient and increasing distress). Comparison between the two timepoints revealed that 74 proteins were downregulated and 15 proteins were upregulated at T4 relative to T1 (Figure 5b). Principal component analysis (PCA) was performed to visualize changes in the proteomic profile and assess clustering between T1 and T4 (Figure 5c). Samples from T1 and T4 largely overlapped, with no clear separation between timepoints and substantial overlap of the 95% confidence intervals, suggesting only minor differences in the global proteomic profiles. Although the T1 group showed slightly greater dispersion, indicating higher variability, overall proteomic profiles remained largely stable across timepoints. A volcano plot (Figure 5d) identified differentially expressed proteins between T4 and T1, with Interferon gamma (IFN-γ), Interleukin (IL)-10 and Leukaemia inhibitory factor (LIF), showing significantly lower expression at T4 (Figure 5e).

No significant differences in inflammatory or immune-related proteins were observed between distress groups (Figure 5f), and no linear correlation was found between protein concentrations and self-reported distress levels. However, CXCL1 showed a significant positive correlation with depression scores (*p* < 0.0001, R^2^ = 0.2201; Figure 5g), and IL-7 showed a significant negative correlation with anxiety scores (*p* < 0.0001, R^2^ = 0.1237; Figure 5h). Both associations were of low-to-moderate strength.

Analyses of the effects of age, sex, cause of injury, and medication use are presented in Supplementary Figures S7 and S8. Principal component and differential expression analyses according to age and sex showed no major batch effects. In contrast, the comparison between traumatic and non-traumatic SCI cases identified several significantly differentially expressed cytokines and immune markers, with HAVCR1, FGF21, and IL17A showing lower expression, whereas IL4 showed higher expression in the non-traumatic group. Analysis of medication use revealed a small effect of NSAID use, with higher IL5 expression.

To account for these differences, we performed an exploratory longitudinal analysis (T1 vs. T4) of protein expression adjusted for medication use and cause of injury. This yielded a similar pattern of results, including a significant decrease in LIF at discharge (Supplementary Figures S9 and S10). IFN-γ and IL-10 also showed lower expression at T4, with log2 fold changes below −0.5 (Supplementary Table S4).

## 3. Discussion

This pilot feasibility study explored the longitudinal relationship between psychological distress and biological markers of stress and aging in individuals with spinal cord injury (SCI) during their first inpatient rehabilitation following injury. Although accelerated aging in SCI remains a plausible hypothesis, a better understanding of the biological mechanisms that may underlie the relationship between psychological distress and recovery after SCI is needed to improve preventive and therapeutic strategies.

Psychological assessments showed that self-reported distress and depressive symptoms significantly decreased by the end of rehabilitation, whereas anxiety levels showed no meaningful change. Despite this overall improvement, 22% of participants continued to report elevated distress at discharge, and distress levels were moderately positively correlated with both depression and anxiety scores. These findings align with previous studies showing that approximately 70% of individuals with SCI exhibit relatively favorable psychological adaptation profiles at discharge, whereas the remaining 30% experience higher levels of depression, anxiety, and distress accompanied by low life satisfaction [10].

To better understand individual differences in distress directional change patterns during rehabilitation, participants were categorized into four groups based on their longitudinal distress profiles: high chronic and low distress groups, characterized by stable distress levels across both timepoints, transient and increasing distress groups, characterized by decreasing and increasing distress at discharge, respectively. The presence of increasing and high chronic distress patterns suggests that not all individuals follow a linear recovery course, and that some may experience persistent or worsening psychological distress, potentially due to reintegration-related anxiety or increased awareness of long-term limitations [11,25]. Other studies have similarly described substantial variability in psychological outcomes following SCI, highlighting the importance of sustained social support during rehabilitation and after discharge [1,5,9,10,26].

To explore whether psychological distress may be associated with biomarkers related to aging in SCI, we assessed longitudinal changes in biomarkers of systemic stress (cortisol), oxidative damage (protein carbonyl content; PCC), and biological aging (telomere length), and compared these changes across the four distress groups.

We observed a significant decrease in PCC levels during rehabilitation. Elevated PCC has been associated with protein damage and dysfunction, which may contribute to cellular impairment, inflammation, and disease progression [27]. Furthermore, previous studies have reported increased oxidative stress after SCI and have proposed that it may contribute to biological processes associated with premature aging [15,22,28]. In the absence of a matched non-SCI control group, our findings reflect relative longitudinal differences only and do not permit conclusions regarding accelerated aging. Instead, they suggest a decrease in PCC during the rehabilitation period, consistent with partial resolution of acute oxidative stress following the initial trauma.

In contrast, no significant longitudinal changes were observed in serum cortisol levels, a widely used biomarker of systemic stress [17], or in telomere length, a recognized marker of cellular aging [13,29]. These findings are consistent with previous literature indicating that cortisol regulation is often altered, but not necessarily elevated, after SCI, likely due to hypothalamic–pituitary–adrenal (HPA) axis dysregulation following neurological trauma [17,30]. Although standardization of blood sampling, with most samples collected in the morning, helped minimize potential confounding due to circadian variation, circulating cortisol was measured only once at each study visit, precluding a diurnal assessment of HPA axis activity.

SCI has been associated with inflammation, oxidative stress, and reduced mobility, all of which have been proposed as potential contributors to age-related biological processes. Assessing telomere length provided an opportunity to explore whether detectable early post-injury changes occurred during rehabilitation. In our study, telomere length did not change during the rehabilitation period. Although telomere length showed a strong negative correlation with age, its stability over the rehabilitation period was expected, given that telomere length changes only minimally over short time intervals in adults [31].

Proteomic analyses revealed a substantial reduction in inflammatory and immune-related proteins after rehabilitation. Specifically, the pro-inflammatory cytokine IFN-γ, together with the anti-inflammatory cytokines IL-10 and LIF, showed significantly lower abundance at discharge. These findings likely reflect attenuation of the acute post-injury inflammatory response during the rehabilitation period [3,32] and are consistent with the possibility that structured rehabilitation environments may contribute to improved immune regulation and overall health [33].

Rehabilitation progress was also reflected in reduced medication use. At baseline, approximately 70% of participants used psychotropic and analgesic medications. By discharge, the use of psychotropic (antiepileptics, psycholeptics, and antidepressants) had decreased to 60%, and analgesic use had declined to 30%, whereas non-steroidal anti-inflammatory drug (NSAIDs) use remained stable at 15% across both timepoints. Previous studies have likewise reported high medication use in SCI populations, particularly for the management of pain and mood symptoms [34,35].

Although an exploratory longitudinal analysis of protein expression adjusted for medication use and cause of injury yielded a similar overall pattern of proteomic results, the independent effects of rehabilitation, medication changes, and spontaneous recovery cannot be isolated within the design of this pilot study. Accordingly, these findings should be considered exploratory, hypothesis-generating, and interpreted with appropriate caution.

Transcriptomic analyses showed significant downregulation of genes associated with adaptive immune function, neural development and apoptosis regulation (IGLV2-14, CELSR1, GSDME, KLHL14, TP73, LINC02805) in individuals with high chronic distress. Consistent with these findings, Gene Set Enrichment Analysis (GSEA) identified reduced activity of pathways involved in adaptive and innate immune responses and complement-mediated inflammation. These findings provide preliminary evidence that persistent psychological distress may be associated with changes in the expression of genes involved in immune-related processes and tissue maintenance. Some of these genes have previously been linked to biological processes associated with aging [8,16,20]. However, given the small subgroup sizes, these differential expression analyses should be considered exploratory. Further studies are needed to determine the biological significance of these associations and to investigate the relationship between persistent psychological distress and aging-related processes following SCI.

Overall, in our study, psychological distress was not linearly associated with inflammatory or immune-related proteins or with systemic biomarkers of stress and aging, highlighting the complex and likely indirect relationship between psychological and biological processes in SCI [12]. Notably, specific inflammatory markers such as CXCL1 and IL-7 showed low-to-moderate correlations with depressive symptoms and anxiety, respectively. CXCL1, a chemokine involved in inflammation and immune cells recruitment to the site of injury [36], has been implicated in neuroinflammation and depression-like behavior in animal models. Previous studies suggest that CXCL1 may influence pro-apoptotic and neuroplasticity-related pathways relevant to mood disturbances [37]. IL-7, which is involved in T-cell homeostasis, has been suggested to play a neuroprotective role and may influence macrophage activity, a key component of post-injury inflammatory and regenerative processes [38]. Its association with anxiety in this study may be consistent with immunoregulatory mechanisms involved in stress responsiveness.

The present study was designed as a pilot and feasibility study, with the primary objective of exploring the feasibility of integrating these data modalities and generating hypotheses for future investigations in larger, independent cohorts. Accordingly, our findings should be considered exploratory and interpreted with caution until replicated in larger cohorts. If confirmed, these findings may help identify inflammatory pathways for further investigation as potential therapeutic targets. Previous work has proposed that immunomodulatory approaches, including cytokine-targeted therapies and neuroimmune interventions, warrant further investigation as potential adjuncts to standard psychological and rehabilitative care following SCI [39].

This study has several limitations that should be considered when interpreting the findings. These include modest sample size and limited statistical power, biological heterogeneity resulting from the inclusion of both traumatic and non-traumatic SCI, potential confounding by concomitant medication use, technical challenges related to hemoglobin contamination of the transcriptomic data, and the absence of a non-SCI control group.

The modest sample size, especially within the longitudinal distress subgroups, limited the statistical power and the stability of the differential expression analyses. In addition, the selection of a subgroup of participants with prototypical distress patterns was not based on a formal clustering approach. Consequently, these subgroup findings require confirmation in larger independent cohorts. However, spinal cord injury is a relatively uncommon condition, and large longitudinal cohorts with paired psychological, transcriptomic, proteomic, and biological aging data are rare.

The inclusion of both traumatic and non-traumatic SCI cases introduced biological heterogeneity. Non-traumatic SCI cases in the SwiSCI cohort may include underlying conditions such as multiple sclerosis, amyotrophic lateral sclerosis, or polyneuropathy. However, given the exploratory nature of this pilot study and the limited sample size, we included both traumatic and non-traumatic SCI cases to retain sufficient sample numbers and to reflect the broader SwiSCI inception cohort.

The potential influence of concomitant medication, particularly psychotropics, analgesics, and anti-inflammatory drugs, on both psychological and biological measures may have acted as a confounding factor. This exploratory model cannot fully separate medication effects from rehabilitation-related or post-injury biological changes. Nevertheless, exploratory longitudinal analyses (T1 vs. T4) of protein expression adjusted for medication use and cause of injury yielded a similar pattern of results, including a significant decrease in LIF at discharge.

Technical issues during sample processing resulted in hemoglobin (HB) contamination of the transcriptomic data, requiring an adaptation of the standard RNA-seq processing workflow to avoid bias in downstream analysis. To mitigate the effect of HB contamination, we extended the standard workflow with additional steps specifically targeting HB-related effects. After filtering and normalization, our quality control assessment showed that residual HB contamination was unlikely to have influenced the observed group differences.

Furthermore, in the absence of a non-SCI control group, our findings should be interpreted within the context of the study population.

Future studies with larger cohorts, standardized sampling protocols, an appropriate healthy control group, and longer follow-up periods are needed to validate and extend these findings.

## 4. Materials and Methods

### 4.1. Study Design

This pilot feasibility study was a nested project within the Swiss Spinal Cord Injury (SwiSCI) Cohort Study [40]. SwiSCI is a prospective observational cohort study that systematically collects demographic, clinical, biopsychosocial data, and biological samples from individuals residing in Switzerland who have been diagnosed with either traumatic or non-traumatic SCI [41].

This project was approved by the Ethikkommission Nordwest- und Zentralschweiz (EKNZ) ethics committee (Project ID: 2017-02060, date of approval: 19 December 2017) and was conducted in accordance with the Declaration of Helsinki and all relevant guidelines and regulations. Written informed consent was obtained from all participants in accordance with the SwiSCI Cohort Study procedures.

### 4.2. Study Population and Sample Preparation

Data were collected from patients enrolled in the SwiSCI Cohort Study between 2016 and 2022, who completed the SwiSCI questionnaire and donated blood samples to the SwiSCI Biobank at baseline (T1) and discharge (T4) from their first rehabilitation (*n* = 120). To characterize different patterns of distress during rehabilitation, we selected 50 individuals from this cohort who represented four distinct distress patterns: “high chronic”, “low”, “increasing”, “transient” distress. The subgroup of 50 individuals was selected from the 120 participants using a structured and predefined approach intended to represent four longitudinal patterns of psychological distress between T1 and T4. Participants were first classified according to their Distress Thermometer scores at T1 and T4: persistently high distress (≥7 at both timepoints), persistently low distress (≤4 at both timepoints), marked increase from low to high distress, and marked decrease from high to low distress. From this preselected pool, samples were retained for transcriptomic analysis only if RNA quality-control criteria were met. The same participants were used for proteomic and biomarker analyses when serum or DNA samples were available in the SwiSCI Biobank.

Statistical comparisons between the full cohort (*n* = 120) and the selected subgroup included in the biological analyses (*n* = 50) were performed to assess representativeness. Continuous variables (e.g., age, time post-injury) were analyzed with the Wilcoxon rank-sum test and categorical variables (e.g., sex, etiology, paralysis) were compared using chi-square tests.

Medications were classified according to the Anatomical Therapeutic Chemical (ATC) classification system as follows: psychotropic drugs, including antiepileptics (N03), psycholeptics (N05; e.g., neuroleptics, anxiolytics, hypnotics, and sedatives), and antidepressants (N06A); analgesics (N02); and non-steroidal anti-inflammatory drugs (NSAIDs; M01A).

Biological samples (serum, RNA and DNA) were provided by the SwiSCI Biobank (Nottwil, Switzerland) at both timepoints T1 and T4. Detailed protocols for sample processing have been described elsewhere [42]. In brief, RNA was extracted from whole blood of 50 participants using the PAXgene Blood miRNA Kit (Qiagen, Steinhausen, Switzerland), and genomic DNA was isolated from whole blood of 49 participants using the GeneJET Genomic DNA Purification Kit (Thermo Scientific, Schlieren Zurich, Switzerland). Serum samples were obtained from 48 participants using S-monovette Z clotting factor (Sarstedt, Sevelen, Switzerland). One DNA sample and two serum samples were not available from the SwiSCI Biobank. All extracted samples were stored at −80 °C until analysis.

### 4.3. Individual Distress Thermometer, Depression and Anxiety Score

The Distress Thermometer was used as part of the SwiSCI questionnaire to assess psychological distress. This self-report instrument asks respondents to rate their perceived level of distress on a visual analogue scale ranging from 0 to 10, where a score of 0 reflects a state of complete calm (“no distress”) and 10 indicates “extreme distress’’ [10,43]. The Hospital Anxiety and Depression Scale (HADS) consists of 14 items, divided into two subscales (anxiety and depression), each yielding a score ranging from 0 to 21. A subscale score greater than 8 indicates clinically relevant levels of anxiety or depression [10,44,45].

### 4.4. Stress and Aging Biomarkers

#### 4.4.1. Cortisol

Serum cortisol concentrations were measured in patients at both timepoints T1 and T4 using the DetectX^®^ Cortisol Enzyme Immunoassay kit (Arbor Assays cat. K003-H1, LubioScience GmbH, Zurich, Switzerland). Samples were diluted 1:100 in accordance with the manufacturer’s instructions. Cortisol levels were quantified at a wavelength of 450 nm using a multi-mode plate reader (BioTek Synergy HTX, Agilent Technologies (Schweiz) AG, Basel, Switzerland). Most serum samples were collected in the morning (between 6:45 and 9:30, *n* = 89; 11:00–12:00, *n* = 3; 14:00–15:00, *n* = 2; unknown, *n* = 2).

#### 4.4.2. Protein Carbonyl Content

Serum Protein Carbonyl Content (PCC) was assessed in patients at both T1 and T4 with fluorometric PCC Assay kit (Abcam cat. ab235631, LubioScience GmbH, Zurich, Switzerland) and normalized to total serum protein concentrations, which were determined using the Nanodrop spectrophotometer (Thermo Scientific, Schlieren Zurich, Switzerland).

#### 4.4.3. Telomere Length

Telomere length was measured in genomic DNA using the Absolute Human Telomere Length Quantification qPCR Assay Kit (ScienCell, Brunschwig, Basel, Switzerland). DNA (5 ng), quantified with Qubit^®^ dsDNA BR Assay Kit (Thermo Scientific, Schlieren Zurich, Switzerland), was amplified in a PCR reaction solution containing primers targeting telomere sequences. A 100 bp-long region on human chromosome 17 served as a single-copy reference (SCR) for normalization. The Comparative ΔΔCq method was applied to quantify telomere length in target samples based on a reference genomic DNA sample with known telomere length.

### 4.5. RNA Sequencing and Transcriptome Analysis

RNA sequencing was performed on samples from 50 selected individuals with SCI at T1 and T4 by Functional Genomics Center Zurich (FGCZ) Zurich. RNA quality was assessed using Qubit^®^ (1.0) Fluorometer (Life Technologies, Carlsbad, CA, USA) and Bioanalyzer 2100 (Agilent, Waldbronn, Germany). Only samples with 260 nm/280 nm ratios between 1.8 and 2.1 and 28S/18S ratios of 1.5–2 were processed further. Total whole-blood RNA (100–1000 ng) was ribo-depleted using Ribo Zero Gold (Epicentre^®^, Madison, WI, USA). Sequencing was performed on an Illumina HiSeq 2500 platform (paired-end 2 × 101 bp or single-end 100 bp) using the TruSeq SBS Kit v4-HS (Illumina, San Diego, CA, USA). Transcriptome analysis was performed using the nf-core RNAseq pipeline (v3.9) and genome assembly GRCh38.p13 (GENCODE v41). Quality control with FastQC [46] revealed hemoglobin contamination due to incomplete depletion. To address this, samples were re-sequenced to increase overall coverage, and next to the standard library size normalization and filtering of genes with low counts (<5), we excluded genes associated with “ERYTHROCYTE”, “BLOOD”, “ERYTHROBLAST”, and “RIBOSOME” gene sets from MSigDB v2022.1 [47], removed extreme outliers (expression > 3× interquartile range [IQR]), and filtered genes contributing to hemoglobin-related batch effects. While this filtering removes a larger fraction of reads in highly contaminated samples, all datasets were subsequently normalized, ensuring comparability across samples (Supplementary Figure S11). We performed comprehensive quality control, including assessment of sequencing depth, gene body coverage, and overall library complexity. All samples met established quality thresholds and were retained for downstream analysis. To further assess potential confounding, we tested whether HB levels were associated with stress groups or timepoints. No significant association was observed between hemoglobin content and psychological distress levels at each timepoint (one-way ANOVA: *p* = 0.892 for stress groups), indicating that HB contamination is unlikely to systematically bias group comparisons (Supplementary Figures S12 and S13).

Differential expression was modelled with a negative binomial generalized linear model (GLM), as implemented in the DESeq2 package [48].

Both T1 and T4 samples were included as separate observations in the design formula (~condi_stress_groups), and contrasts were constructed to compare the combined T1 and T4 samples between the distress groups. Patient ID was not included in the design because each participant belonged to a single distress group, resulting in perfect confounding between patient and group and a non-full-rank design matrix that could not be fitted by DESeq2. Because both timepoints showed consistent directionality of effects, combining them at the contrast level was considered a pragmatic approach to increase statistical sensitivity in this exploratory setting. Consequently, repeated measurements from the same individual were not explicitly modelled, and were therefore treated as independent observations, increasing the nominal sample size (28 samples per group instead of 14 individuals).

False discovery rate correction was applied using the Benjamini–Hochberg (BH) method [49], genes with an adjusted *p* < 0.01 and an absolute log_2_ fold change ≥ 1 were considered differentially expressed. All statistical analyses were performed using the R environment for statistical computing (version 4.0.3) with Bioconductor [50] and dedicated packages.

### 4.6. Proteomic Analyses

Serum proteins of SCI patients were measured using Olink Targeted Proteomics (Olink Proteomics AB, Uppsala, Sweden). Eighty-nine proteins were quantified with two predefined Olink Targeted 48 panels (Cytokines and Immune Surveillance), according to the manufacturer’s instructions. Results were reported as absolute abundances in pg/mL (Olink NPX Signature software v. 2.0.2, Olink Proteomics AB, Uppsala, Sweden).

### 4.7. Statistical Analyses

Results were analyzed using R (version 4.3.3). For the statistical analysis comparing the two timepoints (T1–T4) and the four distress groups, we applied a paired Wilcoxon signed-rank test for non-normally distributed data, accounting for repeated measurements from the same individuals (T1 and T4 samples were not pooled; patient ID was included as a random effect in the mixed-effects model). *p*-values were adjusted for multiple testing using the Benjamini–Hochberg procedure [51]. Differentially expressed genes and proteins were visualized in a volcano plot (ggplot2 v3.5.0), with an absolute log_2_ fold change ≥ 1 and adjusted *p* < 0.01. Principal component analysis (PCA) was used to explore patterns of variation between timepoints and stress groups. All variables, from proteome and transcriptome, were scaled to unit variance prior to PCA to ensure comparability. PCA was carried out using the prcomp (v4.5.1) function in R, and the proportion of variance explained by each principal component was calculated. The PCA scores were visualized in a two-dimensional plot (PC1 vs. PC2) with ggplot2, with samples colored by timepoint and 95% confidence ellipses drawn around each group. Box plots, schematic representations and figures were generated using BioRender (https://www.biorender.com/, accessed on 3 October 2025). Pearson correlation coefficient was used to measure the strength and direction of the linear relationship between two continuous variables. The coefficient ranges from −1 (perfect negative linear correlation) to +1 (perfect positive linear correlation), with 0 indicating no linear relationship.

Quality control and technical performance of proteomic dataset (Supplementary Figures S2–S6) was assessed using deviation of Olink internal controls from the run median. Protein detection status was determined through presence of absolute abundance counts and was successful for all proteins except IFNA2, excluded from the PCA and differential expression analyses. Repeatability was assessed using inter- and intra-run coefficients of variation (CVs) based on triplicate Sample Control measurements. Principal component analysis (PCA) and differentially expressed proteins were analyzed for differences in age, sex, injury cause (traumatic and non-traumatic) and medication use (Supplementary Figures S7 and S8). Exploratory longitudinal analyses of protein expression were performed using linear mixed-effects models adjusted for pharmacological (three binary covariates based on Anatomical Therapeutic Chemical [ATC] codes; 1 = present, 0 = absent), and clinical (SCI cause) covariates (Supplementary Figures S9 and S10).

## 5. Conclusions

This exploratory study demonstrates the feasibility of integrating multi-omics profiling with longitudinal assessments of psychological distress during early SCI rehabilitation. The findings indicate decreases in self-reported distress and depressive symptoms, markers of oxidative damage (PCC), and inflammatory protein expression (i.e., LIF, IFN-γ and IL-10) during rehabilitation, alongside persistent psychological distress in a substantial subgroup of participants. Lower expression of genes involved in adaptive immunity, neural development and apoptosis was observed in individuals with high chronic distress. Given the pilot nature of the study, these findings require validation in larger, independent cohorts with longer follow-up periods to determine the biological significance of the observed biomarker associations, clarify the biological mechanisms that may underlie the relationship between psychological distress and recovery after SCI, and assess whether specific patient subgroups may benefit from targeted psychobiological and immunomodulatory interventions.

## Figures and Tables

**Table 1:** Clinical characteristics of the study participants. Quantitative variables are presented as median (IQR), and qualitative variables as relative frequencies (%). *p*-values indicate comparison between the two groups. Categorical variables were analyzed using the chi-square test, and continuous variables using the Wilcoxon test.

Variables	SwiSCI Patients SwiSCI Dataset	SwiSCI Patients Biological Analysis	*p*-Value
Sample size	120	50	
Age years [range]	52 (29) [17–86]	54 (30) [20–86]	0.70
Sex			0.78
Males	77%	80%	
Females	23%	20%	
Etiology			0.82
Traumatic	69%	66%	
Non-traumatic ^1^	31%	34%	
Paralysis			0.17
Paraplegic	61%	48%	
Tetraplegic	39%	52%	
Days post injury T1			
Questionnaire	43 (40)	44 (41)	0.74
Biobank sample		47 (20)	
Days post injury T4			
Questionnaire	176 (84)	175 (113)	0.67
Biobank sample		177 (107)	
Medications T1			
Psychotropic drugs ^2^	72%	76%	0.69
Analgesics	68%	68%	1
NSAIDs	15%	14%	1
Medications T4			
Psychotropic drugs ^2^	60%	66%	0.57
Analgesics	32%	40%	0.38
NSAIDs	16%	14%	0.94

^1^ SCI was attributed to underlying conditions such as multiple sclerosis, amyotrophic lateral sclerosis, or polyneuropathy. ^2^ Antiepileptics, psycholeptics, and antidepressants.

## Data Availability

This study was conducted as a SwiSCI nested project within the prospective, multicenter Swiss Spinal Cord Injury Cohort Study (SwiSCI). The datasets generated and/or analyzed during the current study contain sensitive human omics and linked clinical data and are therefore not publicly available. In accordance with the SwiSCI governance framework, requests for access to the minimum de-identified dataset required to verify the methods and findings reported in this article may be submitted to the SwiSCI Study Center (contact@swisci.ch) and will be reviewed in line with project-governance procedures, ethics approval, participant consent, and applicable Swiss legal and data-protection requirements. Further information on the SwiSCI Study is available here (SwiSCI—Home).

## Supplementary Materials

The following supporting information can be downloaded at: https://www.mdpi.com/article/10.3390/ijms27156574/s1.

## Abbreviations

The following abbreviations are used in this manuscript:

SCI	Spinal Cord Injury
SwiSCI	Swiss Spinal Cord Injury Cohort Study
PCC	protein carbonyl content
IFN-γ	Interferon gamma
IL	Interleukin
LIF	Leukaemia inhibitory factor
CXCL1	C-X-C motif chemokine ligand 1
HPA	hypothalamic–pituitary–adrenal
NSAIDs	non-steroidal anti-inflammatory drugs
GSDME	Gasdermin E
KLHL	Kelch-like
PCA	Principal Component Analysis
