# Supplementary material for: Psychological Distress and Biological Stress Markers After Spinal Cord Injury: A Longitudinal Multi-Omics Pilot Study
Source: Int J Mol Sci. 2026 Jul 23;27(15):6574. doi: 10.3390/ijms27156574 (PMC13465420; doi:10.3390/ijms27156574)
Supplement: Supplementary file 1 [file ijms-27-06574-s001.zip › ijms-4395023-supplementary.pdf]

Psychological Distress and Biological Stress Markers After Spinal Cord Injury: A Longitudinal Multi-Omics Pilot Study

Simona Capossela [1], Alessandro Bertolo [1,2,*], Alexander Stacul [1], Franziska Singer [3,4], Claudio Peter [5], Xavier Jordan [6], Margret Hund-Georgiadis [7], Björn Zörner [8] and Jivko Stoyanov [1,2]

1. SCI Population Biobanking and Translational Research, Swiss Paraplegic Research, 6207 Nottwil, Switzerland; simona.capossela@paraplegie.ch (S.C.)
2. Institute of Social and Preventive Medicine, University of Bern, 3012 Bern, Switzerland
3. NEXUS Personalized Health, ETH Zurich, 8092 Zurich, Switzerland
4. SIB Swiss Institute of Bioinformatics, 1015 Lausanne, Switzerland
5. Department of Health, ZHAW Zurich University of Applied Sciences, 8400 Winterthur, Switzerland
6. Clinique Romande de Réadaptation, 1951 Sion, Switzerland
7. REHAB Basel, 4055 Basel, Switzerland
8. Swiss Paraplegic Center, 6207 Nottwil, Switzerland

* Correspondence: alessandro.bertolo@paraplegie.ch; Tel.: +41-41-939-6633; Fax: +41-41-939-6640

Supplementary Information

Figure S1. Distress directional change patterns across all timepoints. Gray lines trace individual subject profiles across four timepoints (T1-T2-T3-T4). Missing intermediate data points are bypassed due to overlap between timepoints. For all groups at timepoint T1 and T4 we have a complete dataset (increasing n=8; low n=14; transient n=14; high chronic n=14). For the increasing distress group n=4 at T2 and n=1 at T3; for the low distress group n=8 at T2 and n=7 at T3; for the transient distress group n=9 at T2 and n=9 at T3; and for the high chronic distress group n=8 at T2 and n=5 at T3.

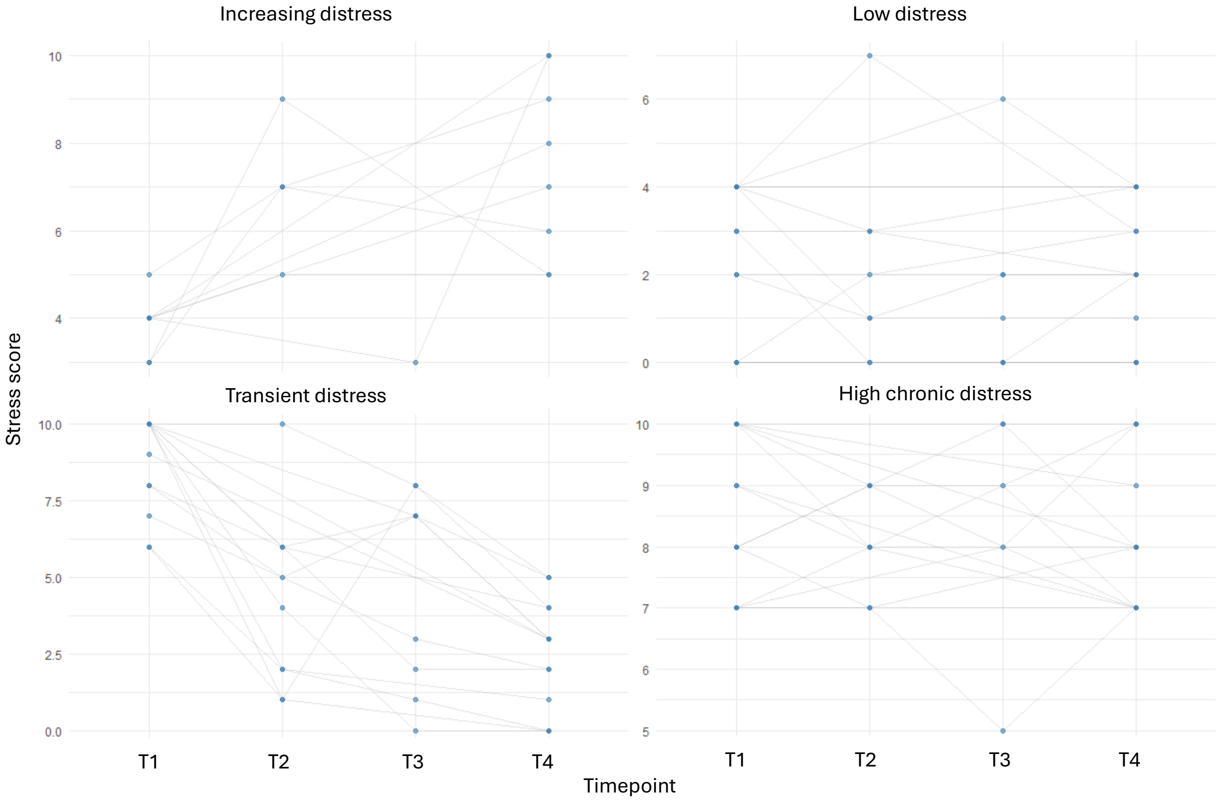

Figure S2. Quality Control Overview for the Target 48 Cytokine, and Target 48 Immune Surveillance panel.

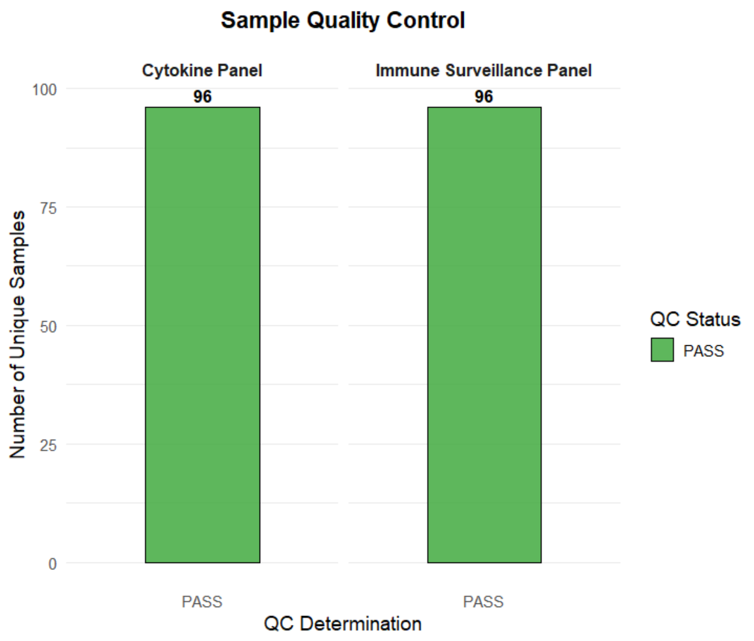

Figure S3. Technical Deviation Thresholds for the Target 48 Cytokine (blue) and Target 48 Immune Surveillance (violet) panel; dotted line = ± 0.3 threshold.

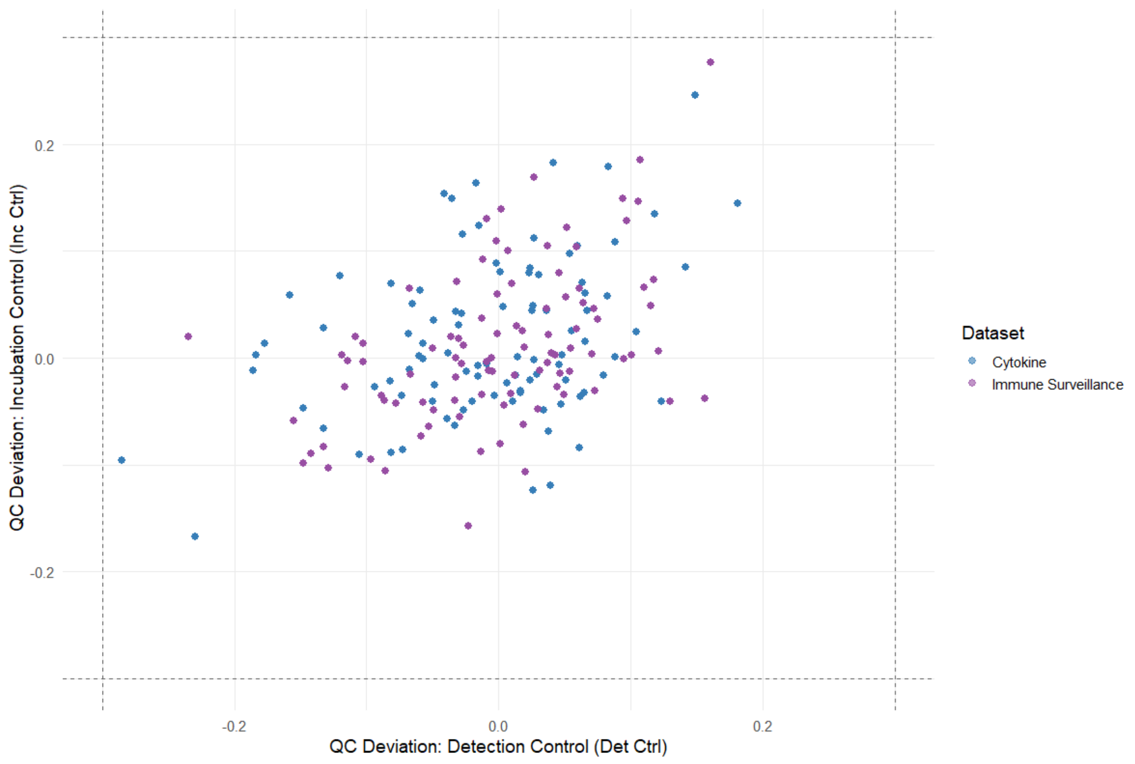

Figure S4. Protein Quantification Status for the Target 48 Cytokine panel.

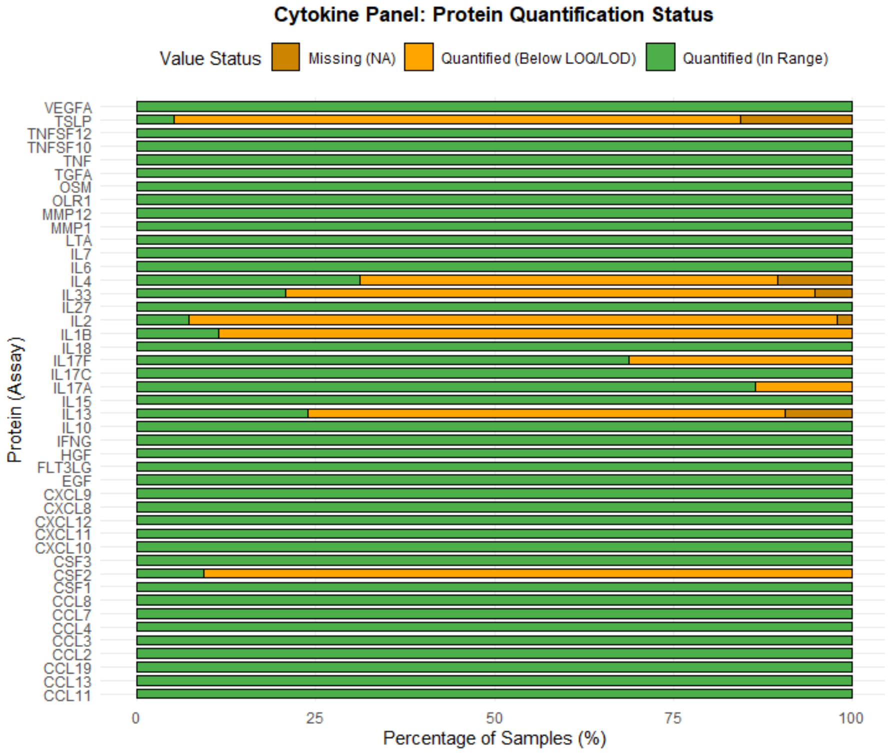

Figure S5. Protein Quantification Status for the Target 48 Immune Surveillance panel.

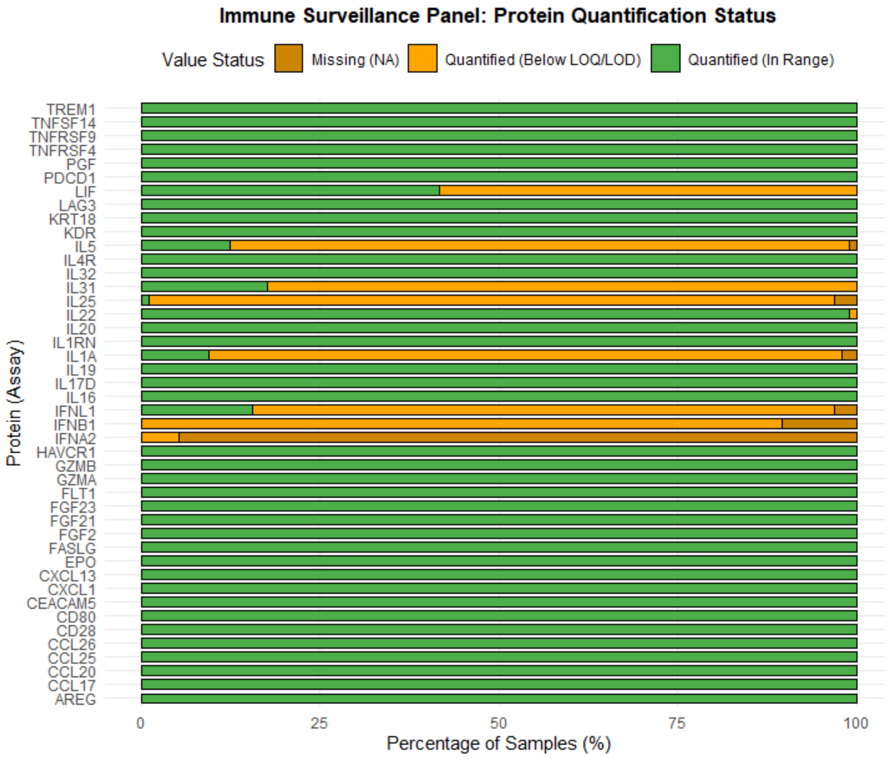

Figure S6. Inter- and Intra-plate Coefficient of Variation (CV) measure for repeatability of protein biomarker assays in Target 48 Cytokine and Target 48 Immune Surveillance panel.

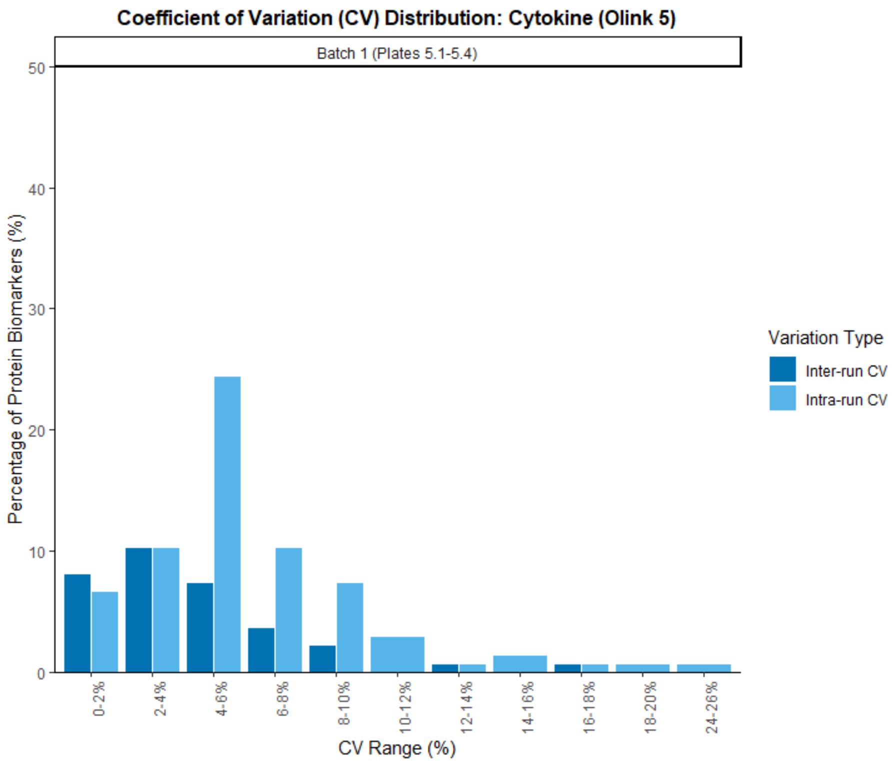

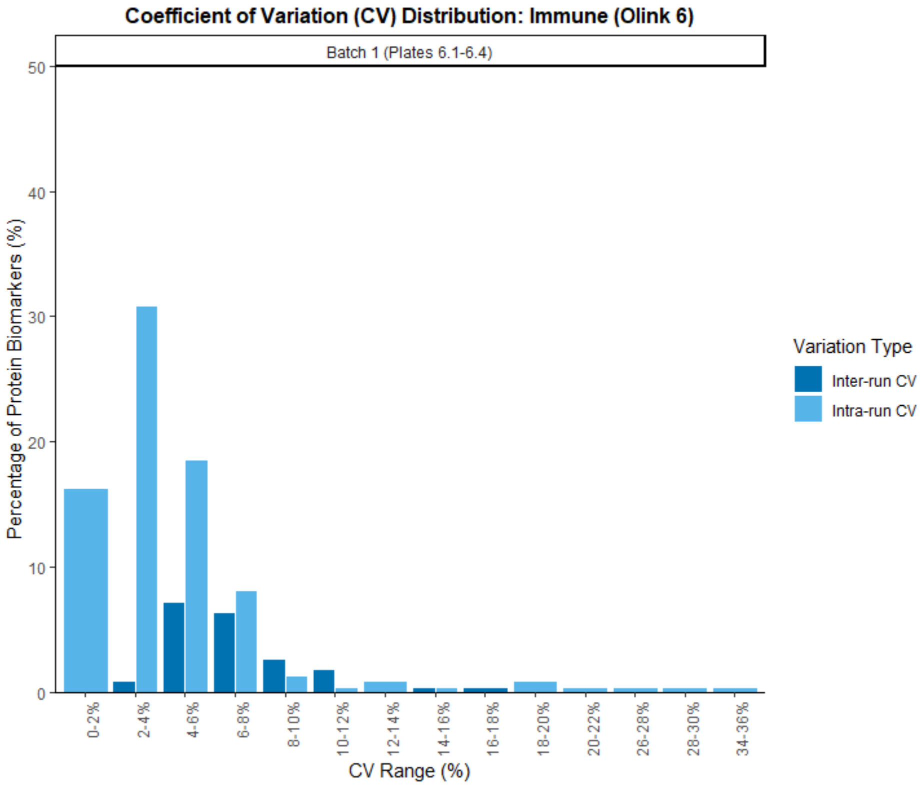

Figure S7. PCA for Target 48 Cytokine and Target 48 Immune Surveillance related to age, sex, SCI cause (T: traumatic and NT: non-traumatic) and medication use (Psychotropics, Analgescis, NSAIDs).

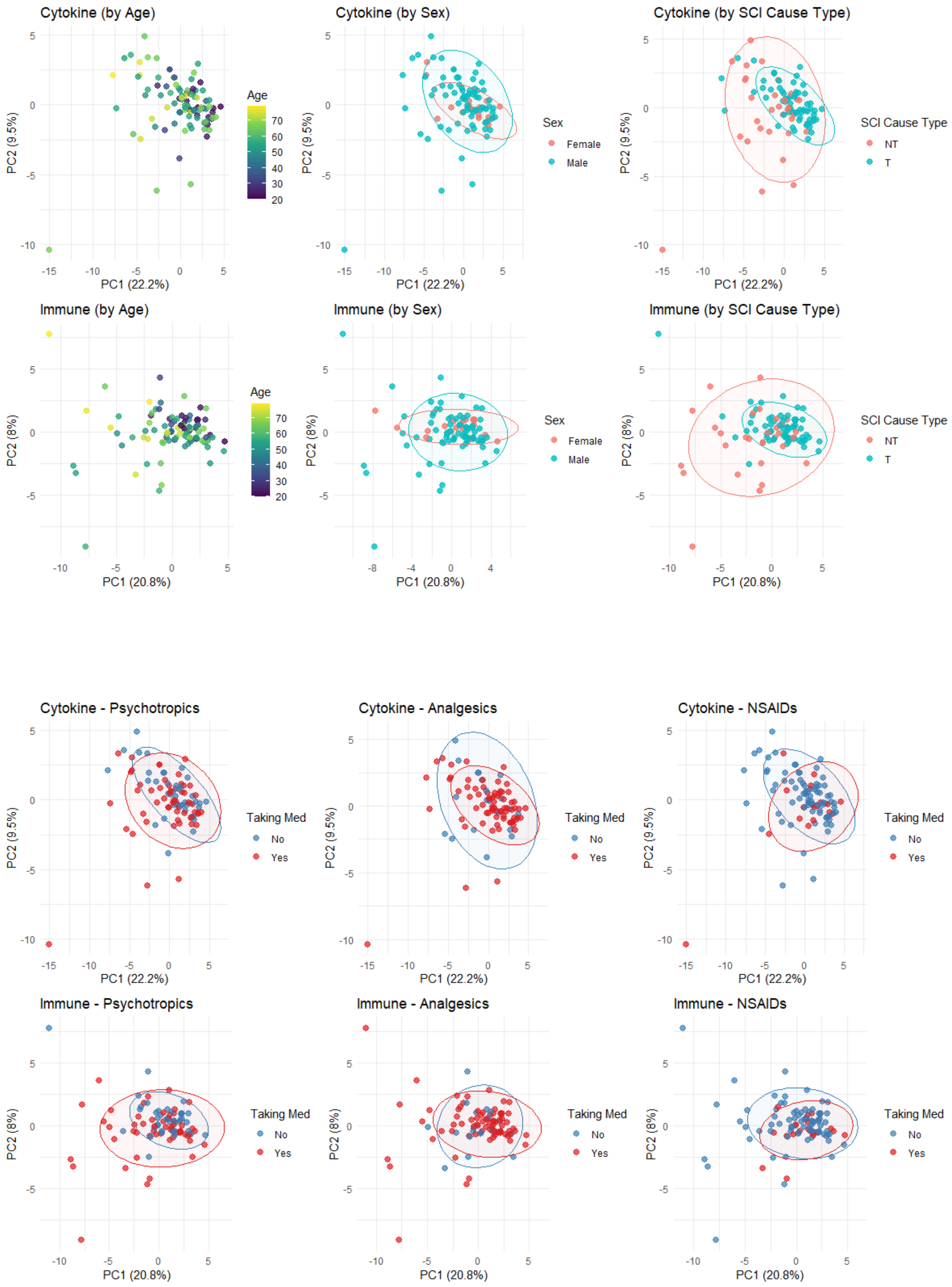

Figure S8. Differentially expressed proteins for Target 48 Cytokine and Target 48 Immune Surveillance related to age, sex, SCI cause, and medication (psychotropics, analgesics, NSAIDs).

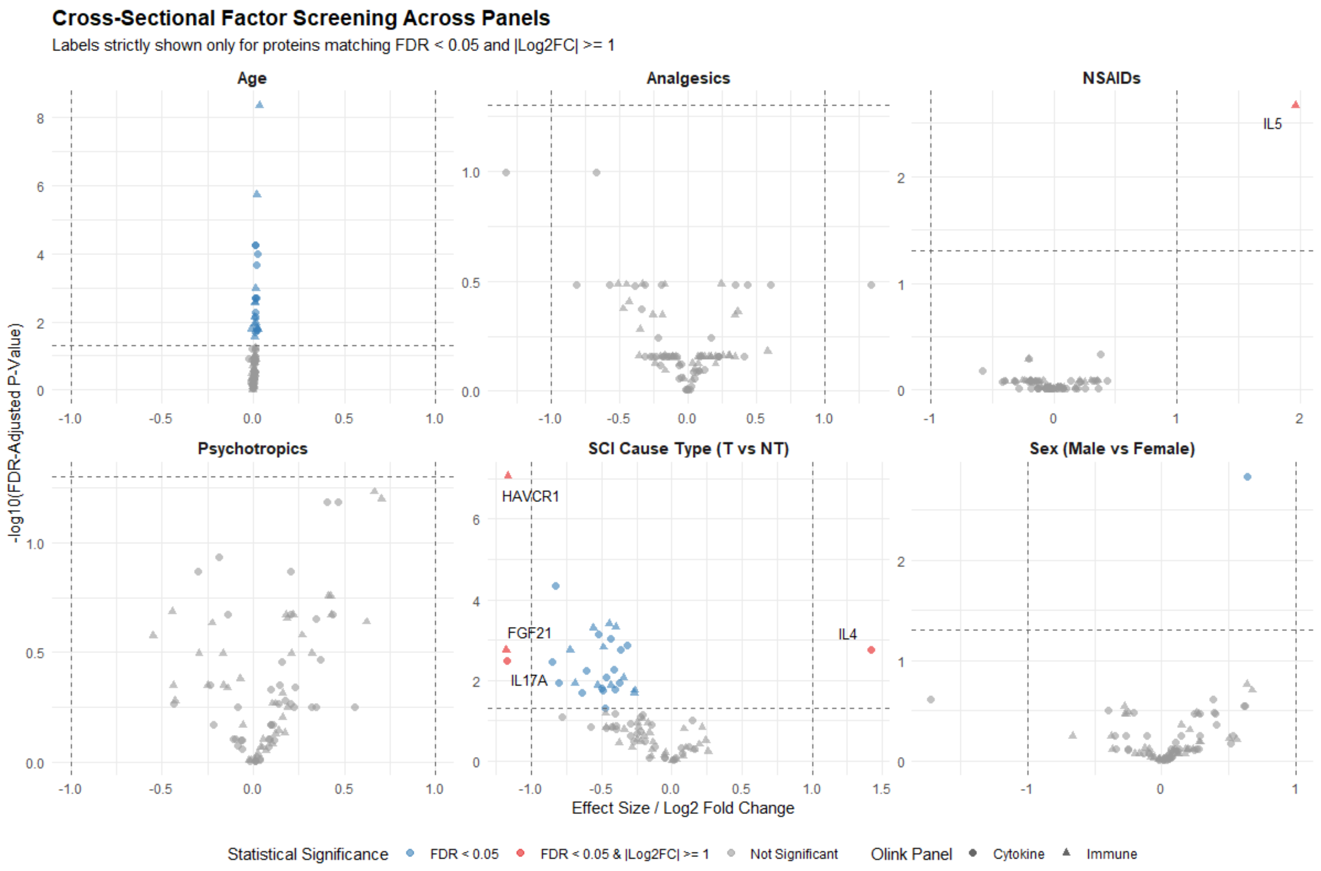

Figure S9. Volcano plot of longitudinal protein changes across the Olink Cytokine and Immune Surveillance panels. Linear mixed-effects models were fitted for each protein using timepoint (T4 versus T1), SCI cause type, psychotropic, analgesic and NSAIDs medication use as fixed effects and participant ID as a random effect. The x-axis represents the estimated log2 fold change (T4 relative to T1), and the y-axis represents the −log10 FDR-adjusted P value. Proteins with FDR-adjusted P < 0.05 and absolute |log2 fold change| ⩾ 1 are highlighted and labelled.

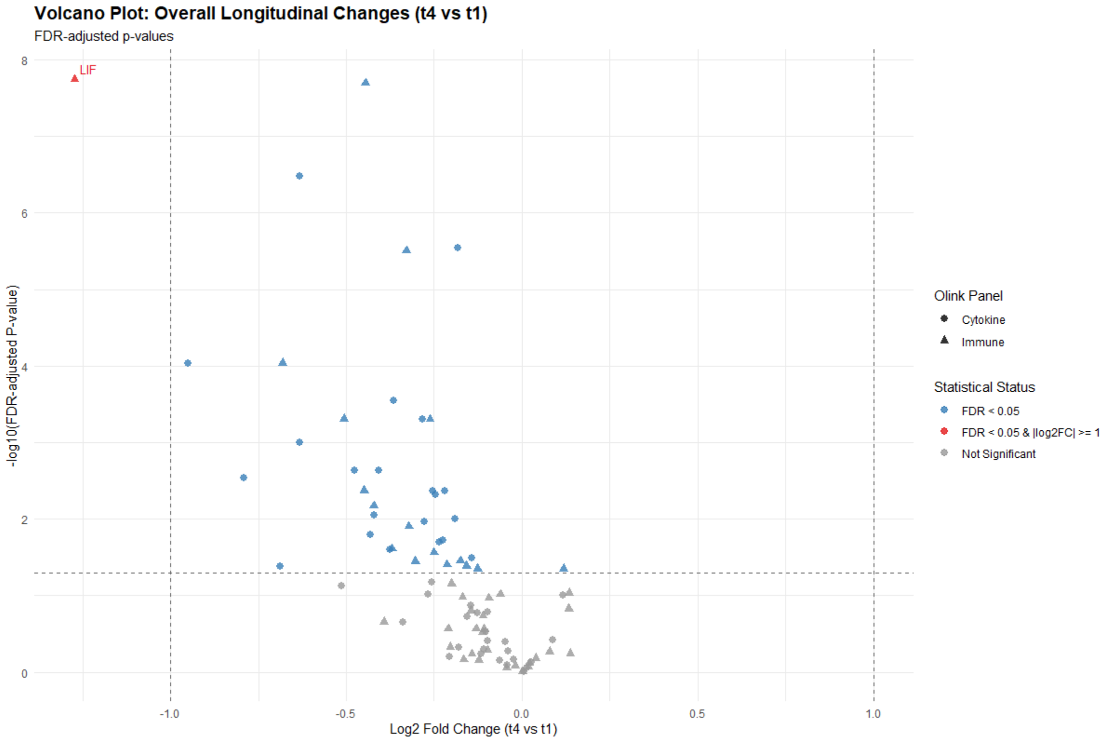

Figure S10. Box plots showing serum Log2 Protein Abundance of LIF at T1 and T4.

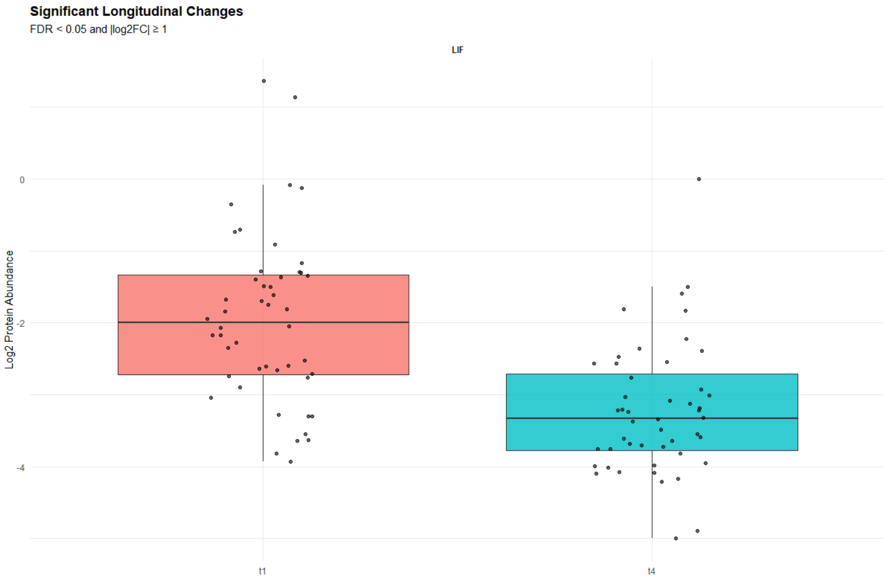

Figure S11. Sample library size (a) before and (b) after normalization. Hemoglobin (HB) groups were defined based on fraction of HB-related reads: low being less than 20% and high being greater than 50% of the total count.

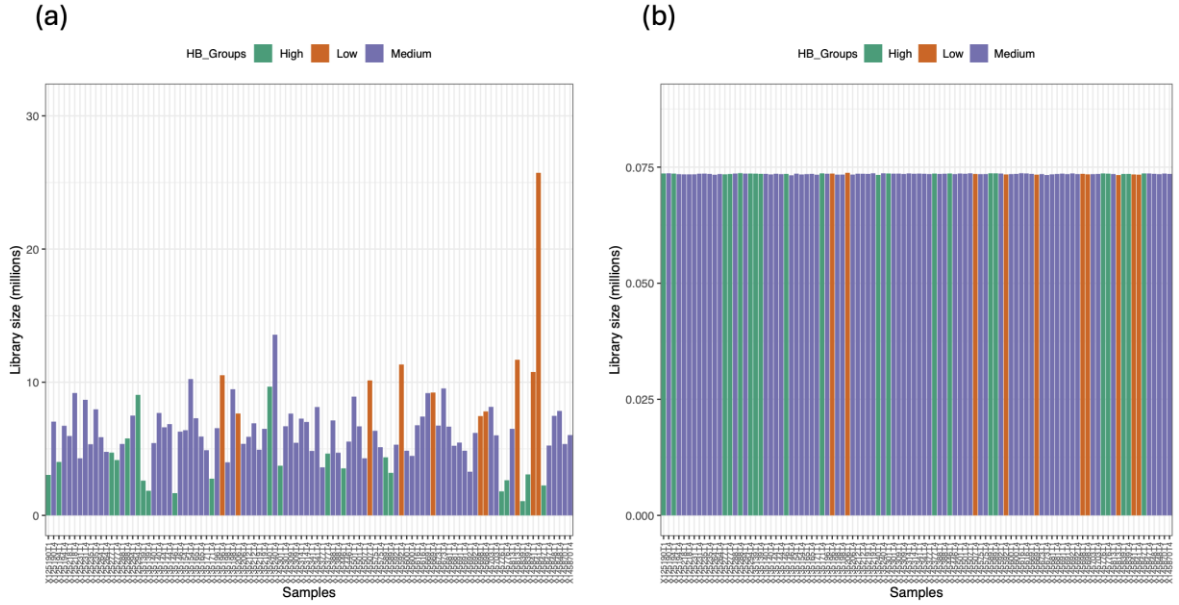

Figure S12. PCA with samples coloured by hemoglobin contamination category (a) before and (b) after contamination removal. Hemoglobin (HB) groups were defined based on fraction of HB-related reads: low being less than 20% and high being greater than 50% of the total count.

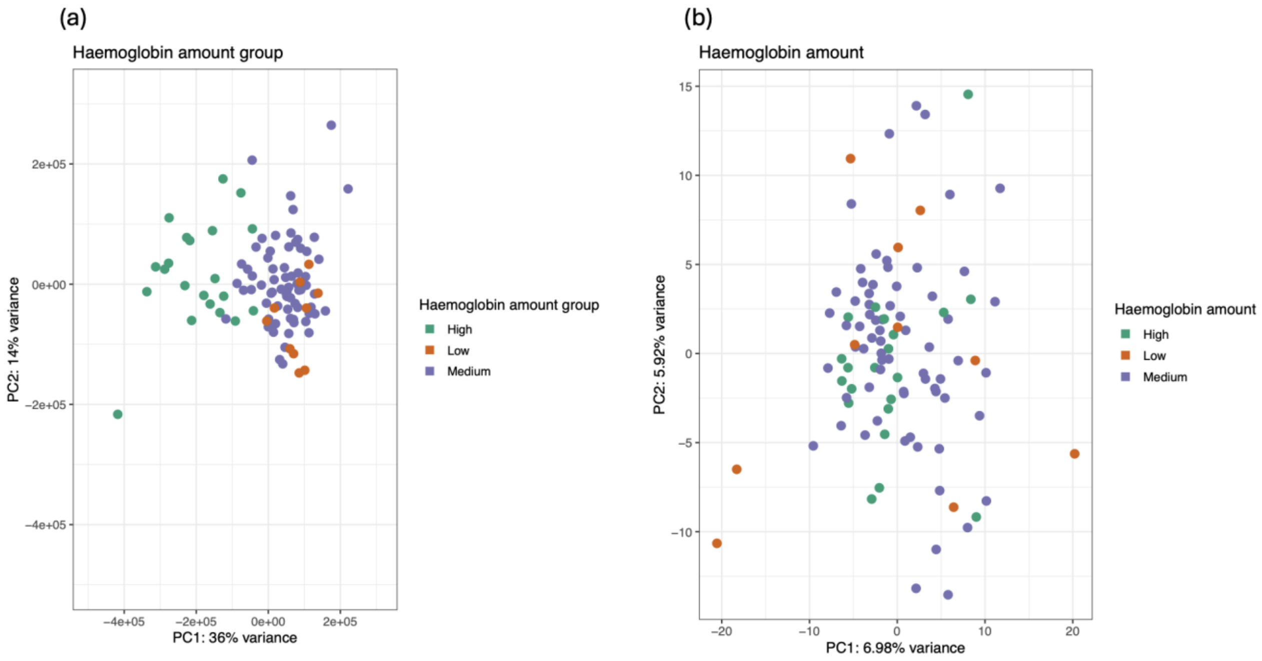

Figure S13. (a) Violin plot showing the hemoglobin (HB) amount (in percent of total reads) per stress group. (b) Hemoglobin amount in percent at each timepoint and reported stress level.

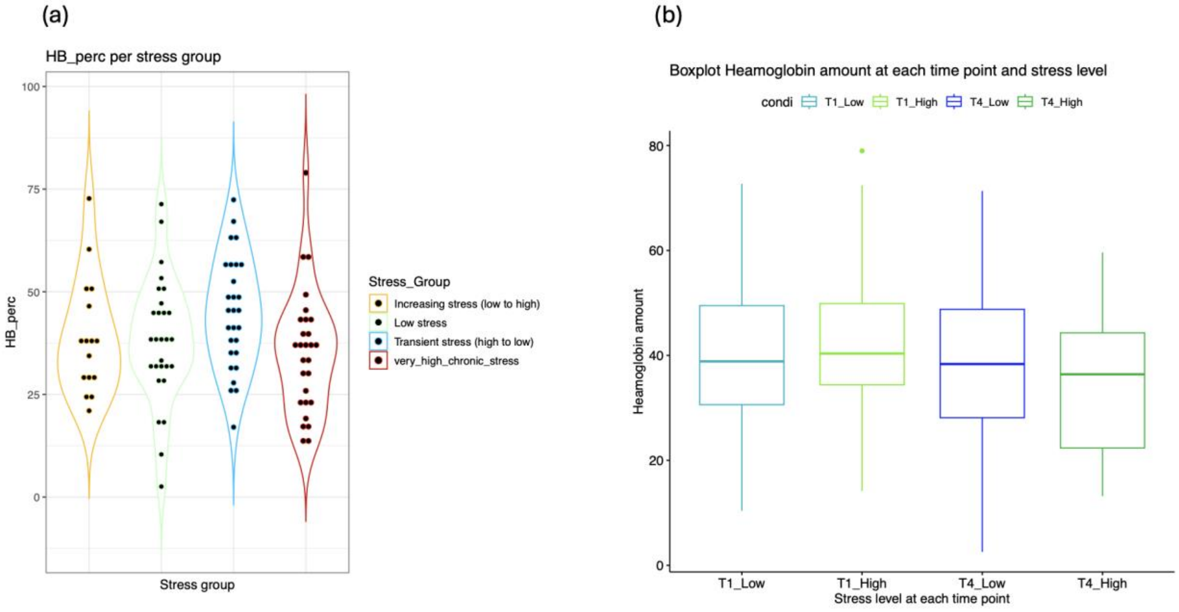

Table S1. Comparison between the high chronic and low distress groups identified six significantly downregulated genes in the high chronic distress group.

SYMBOL	Base Mean	logFC	lfcSE	stat	pvalue	padj	direction
KLHL14	150.9344	-1.22066	0.208021	-5.86795	4.41E-09	8.11E-05	signif & strong down
GSDME	32.49524	-1.2201	0.218992	-5.57143	2.53E-08	0.000404	signif & strong down
LINC02805	13.21865	-1.10073	0.198791	-5.5371	3.08E-08	0.000424	signif & strong down
TP73	21.11248	-1.15079	0.216291	-5.32059	1.03E-07	0.001246	signif & strong down
IGLV2-14	2291.023	-1.03715	0.197226	-5.25869	1.45E-07	0.001311	signif & strong down
CELSR1	241.8142	-1.13592	0.219045	-5.18578	2.15E-07	0.001482	signif & strong down

Table S2. Reactome pathway and Gene Set Enrichment Analysis (GSEA) of downregulated genes in the high chronic distress group compared with the low distress group.

Direction	name	NGenes	FDR
Down	REACTOME ANTIGEN ACTIVATES B CELL RECEPTOR BCR LEADING TO GENERATION OF SECOND MESSENGERS	26	4.66E-06
Down	REACTOME CD22 MEDIATED BCR REGULATION	23	6.06E-06
Down	REACTOME SCAVENGING OF HEME FROM PLASMA	23	1.36E-05
Down	REACTOME FCGAMMA RECEPTOR FCGR DEPENDENT PHAGOCYTOSIS	46	0.000017
Down	REACTOME PARASITE INFECTION	39	1.97E-05
Down	REACTOME ROLE OF PHOSPHOLIPIDS IN PHAGOCYTOSIS	29	2.63E-05
Down	REACTOME CREATION OF C4 AND C2 ACTIVATORS	23	5.48E-05
Down	REACTOME INITIAL TRIGGERING OF COMPLEMENT	24	5.84E-05
Down	REACTOME COMPLEMENT CASCADE	26	0.000101
Down	REACTOME FCGR ACTIVATION	24	0.000134
Down	REACTOME FCERI MEDIATED MAPK ACTIVATION	27	0.000136
Down	REACTOME BINDING AND UPTAKE OF LIGANDS BY SCAVENGER RECEPTORS	31	0.000197
Down	REACTOME ROLE OF LAT2 NTAL LAB ON CALCIUM MOBILIZATION	25	0.000242
Down	REACTOME FCERI MEDIATED CA 2 MOBILIZATION	32	0.000308
Down	REACTOME SIGNALING BY THE B CELL RECEPTOR BCR	43	0.000674
Down	REACTOME LEISHMANIA INFECTION	74	0.000804
Down	REACTOME FCGR3A MEDIATED IL10 SYNTHESIS	35	0.00188
Down	REACTOME ANTI INFLAMMATORY RESPONSE FAVOURING LEISHMANIA PARASITE INFECTION	48	0.00231
Down	REACTOME POTENTIAL THERAPEUTICS FOR SARS	50	0.012
Down	REACTOME PLATELET ACTIVATION SIGNALING AND AGGREGATION	45	0.0156
Down	REACTOME FC EPSILON RECEPTOR FCERI SIGNALING	50	0.0228
Down	REACTOME FCERI MEDIATED NF KB ACTIVATION	39	0.0268
Mixed	REACTOME ASSEMBLY OF COLLAGEN FIBRILS AND OTHER MULTIMERIC STRUCTURES	16	0.0289
Mixed	REACTOME FORMATION OF THE CORNIFIED ENVELOPE	20	0.0354
Mixed	REACTOME KERATINIZATION	20	0.0354
Down	REACTOME HEMOSTASIS	152	0.0437

Table S3. Inter-run and Intra-run Coefficient of Variation (CV).

Panel	Type	Mean CV	Median CV
Cytokine	Inter-run CV	4.14	3.69
Cytokine	Intra-run CV	6.16	5.17
Immune Surveillance	Inter-run CV	7.21	6.41
Immune Surveillance	Intra-run CV	4.62	3.62

Table S4: Differentially expressed proteins with FDR-adjusted P < 0.05 in the longitudinal analysis (T4 vs T1).

ASSAY	ESTIMATE T4_VS_T1	STD ERROR	P VALUE	PANEL	ADJUSTED P VALUE	SIG
**LIF**	-1.2731821	0.1579203	2.06E-10	Immune	1.81E-08	FDR < 0.05 & |log2FC| >= 1
**IL4R**	-0.444764	0.0568018	4.57E-10	Immune	2.01E-08	FDR < 0.05
**CCL7**	-0.6323588	0.0915578	1.13E-08	Cytokine	3.32E-07	FDR < 0.05
**CSF1**	-0.1813999	0.0292125	1.30E-07	Cytokine	2.85E-06	FDR < 0.05
**TNFSF14**	-0.3289053	0.0537336	1.76E-07	Immune	3.11E-06	FDR < 0.05
**IL6**	-0.9503614	0.1884597	7.28E-06	Cytokine	9.15E-05	FDR < 0.05
**KRT18**	-0.681025	0.1345109	6.80E-06	Immune	9.15E-05	FDR < 0.05
**CCL3**	-0.3648107	0.0782052	2.59E-05	Cytokine	2.85E-04	FDR < 0.05
**HGF**	-0.2823041	0.0637195	5.60E-05	Cytokine	4.93E-04	FDR < 0.05
**FGF2**	-0.5066086	0.1137154	5.17E-05	Immune	4.93E-04	FDR < 0.05
**CXCL1**	-0.2619643	0.0595662	6.23E-05	Immune	4.98E-04	FDR < 0.05
**IL10**	-0.6325192	0.1521047	1.35E-04	Cytokine	9.89E-04	FDR < 0.05
**CXCL11**	-0.4085597	0.1064284	3.68E-04	Cytokine	2.33E-03	FDR < 0.05
**IL27**	-0.4757632	0.1240085	3.71E-04	Cytokine	2.33E-03	FDR < 0.05
**IFNG**	-0.7918744	0.2115838	4.95E-04	Cytokine	2.91E-03	FDR < 0.05
**IL7**	-0.2189971	0.0611396	8.06E-04	Cytokine	4.27E-03	FDR < 0.05
**EPO**	-0.4492503	0.1256845	8.25E-04	Immune	4.27E-03	FDR < 0.05
**TNF**	-0.2539491	0.0714307	8.74E-04	Cytokine	4.27E-03	FDR < 0.05
**EGF**	-0.246225	0.0704124	1.04E-03	Cytokine	4.81E-03	FDR < 0.05
**IL1RN**	-0.4217422	0.1254401	1.55E-03	Immune	6.80E-03	FDR < 0.05
**CXCL10**	-0.4210862	0.1293042	2.10E-03	Cytokine	8.78E-03	FDR < 0.05
**CCL8**	-0.191188	0.0599024	2.52E-03	Cytokine	1.01E-02	FDR < 0.05
**VEGFA**	-0.278867	0.0884942	2.83E-03	Cytokine	1.08E-02	FDR < 0.05
**AREG**	-0.3219511	0.1043384	3.40E-03	Immune	1.25E-02	FDR < 0.05
**IL1B**	-0.4305998	0.1447371	4.61E-03	Cytokine	1.62E-02	FDR < 0.05
**CXCL8**	-0.2248996	0.077442	5.60E-03	Cytokine	1.89E-02	FDR < 0.05
**CCL4**	-0.2362658	0.0824786	6.22E-03	Cytokine	2.03E-02	FDR < 0.05
**IL22**	-0.3697544	0.1330495	7.81E-03	Immune	2.46E-02	FDR < 0.05
**MMP12**	-0.3767712	0.1365025	8.21E-03	Cytokine	2.49E-02	FDR < 0.05
**CCL17**	-0.2500045	0.0923025	9.40E-03	Immune	2.76E-02	FDR < 0.05
**IL15**	-0.1422745	0.053993	1.14E-02	Cytokine	3.22E-02	FDR < 0.05
**PGF**	-0.1756718	0.0678457	1.28E-02	Immune	3.51E-02	FDR < 0.05
**GZMB**	-0.3032304	0.1180381	1.34E-02	Immune	3.58E-02	FDR < 0.05
**CXCL13**	-0.2135672	0.0847663	1.52E-02	Immune	3.94E-02	FDR < 0.05
**CD80**	-0.1574382	0.0632641	1.64E-02	Immune	4.13E-02	FDR < 0.05
**IL4**	-0.6880971	0.2769844	1.70E-02	Cytokine	4.16E-02	FDR < 0.05
**CCL25**	0.1185505	0.0489598	1.94E-02	Immune	4.49E-02	FDR < 0.05
**FLT1**	-0.1258858	0.0519392	1.93E-02	Immune	4.49E-02	FDR < 0.05
